# 

**Published:** 1843-05-27

**Authors:** 


CONTENTS.

A Course of Clinical Lectures, delivered at the Hôtel Dieu, Paris, for the Session 1842–'43. By A. F. Chomel, M.D. Lecture 8. Iliac Abscess, . . 109

Case 1.—Post-puerperal peritonitis—Consecutive iliac abscess—Spontaneous perforation—Acute peritonitis—Death, . . . . . . . . 109

Case 2.—Abscess in the left iliac fossa—Development of another similar one on the right side—Symptoms of hectic fever—Convalescence, . . . . 110

Case 3.—Iliac phlegmon terminating by resolution, . . . . . . 112

Sulphate of Alumina as an antiseptic, . 112

Case of a needle entering the right breast, and finally lodging in the heart, causing death. By B. F. Leaming, M. D., 112

Suppression of Quackery, . . . . 113

A list of cases discharged from the surgical wards of the Pennsylvania Hospital, during the month of April, 1843. By Ellerslie Wallace, M. D. . . 113

Editorial—Medical Ethics, . . 115

Tubercles of the Serous Membranes, . 117

Delirium Tremens, . . . . . 117

*Retrospect of the Medical Sciences.*

Aneurysm of Abdominal Aorta opening into the lungs, . . . . . 118

Experiments on the Torpedo, . . 118

Stammering, . . . . . . . 118

Remarks on the Calculi in St. George's Hospital. By Dr. Bence Jones, . 119

Urethroplasty, . . . . . . . 120

Pilulæ Hydrarg. Cum Ferro, . . 120

Value of Carbonic Acid with Quinine in Intermittent Fevers, . . . . 120

Revival of the Arts among the Greeks, 120

Cellular cysticercus under the conjunctiva 120

Adulteration of Milk, . . . . . 120

